# The impact of Australian healthcare reforms on emergency department time-based process outcomes: An interrupted time series study

**DOI:** 10.1371/journal.pone.0209043

**Published:** 2018-12-12

**Authors:** Khic-Houy Prang, Rachel Canaway, Marie Bismark, David Dunt, Margaret Kelaher

**Affiliations:** Centre for Health Policy, Melbourne School of Population and Global Health, The University of Melbourne, Melbourne, Victoria, Australia; University of Malta Faculty of Health Sciences, MALTA

## Abstract

**Background:**

In 2011, the Australian government introduced national healthcare reforms aimed at increasing the timeliness and quality of hospital care. The healthcare reforms included, but were not limited to, emergency department (ED) time-based targets, financial incentives, and public performance reporting of hospital data. We sought to evaluate the impact of the national healthcare reforms on ED time-based process outcomes.

**Methods:**

A quasi-experimental study of ED presentations from 2006 to 2016 in the state of Victoria, Australia. Uncontrolled, interrupted time-series analyses were used to evaluate, by hospital peer groups, the effect of national healthcare reforms on: patient wait times for treatment; treatment within recommended time; and patient departure within four hours of arrival in ED.

**Results:**

There were small improvements in ED time-based process outcomes following the introduction of the national healthcare reforms. These occurred in most hospital peer groups immediately and over the longer term, across the various triage categories. The largest improvements occurred in small hospitals and smallest improvements in medium sized hospitals. ED time-based targets, now abolished by the Australian government, were not achieved in any hospital peer groups.

**Conclusions:**

Our findings suggest that national healthcare reforms had the potential to prompt fundamental changes in ED processes leading to significant improvements in ED performances across most hospital peer groups but were generally unable to reach the ED targets imposed nationally. ED performances also varied by hospital peer groups. Attention to ED time-based process outcomes within hospital peer groups may provide insights into hospital practices that could improve the quality and efficiency of ED care.

## Introduction

Increased demands for emergency care due to changes in population growth, an ageing population and increasing prevalence of chronic diseases, have led to overcrowding, increased waiting times and length of stay (LOS) in emergency departments (ED) worldwide [1, 2]. ED crowding and delays are associated with adverse outcomes for patients including increased mortality [3, 4] and length of hospital stay [5, 6], and lowered levels of satisfaction [7, 8].

To ameliorate ED crowding, the United Kingdom introduced a time-based emergency care target in 2004, which stipulated a maximum limit of four hours on the LOS from time of arrival to discharge or transfer to an inpatient ward [9]. Many countries, including Australia [10], New Zealand [11] and some Canadian provinces [12], similarly introduced ED time-based targets for time spent in ED from arrival to departure, ranging from 4 to 8 hours. In Australia, the National Emergency Access Target (NEAT) was introduced in 2011 as part of the National Health Reform Agreement (NHRA) [13] and the National Partnership Agreement on Improving Public Hospital Services [14]. The goal of NEAT was to increase (towards 90% by 2015) the proportion of patients discharged, admitted or transferred to another hospital within four hours of arrival at an ED. Initially, state and territory governments received significant financial incentives from the Australian government when ED targets were achieved; however, these payments ceased in 2014.

Furthermore, the NHRA mandated that all public hospitals report their performance data to the National Health Performance Authority (reporting transferred to the Australian Institute of Health and Welfare (AIHW) in 2016) for public reporting on the MyHospitals website [15]. In contrast, public reporting on the MyHospitals website is voluntary for private hospitals. Other ED performance indicators publicly reported on the MyHospitals website include the proportion of patients seen within clinically recommended triage times, as recommended by the Australasian College for Emergency Medicine [16]. This target was set at 80% across all triage categories. The MyHospitals website allows the public to access national, comparable performance information on hospitals and hospital providers.

Previous studies have demonstrated that the introduction of time-based targets with sanctions (i.e. publicly naming and shaming hospitals) have led to improvement in waiting times for elective care [17, 18]. Similarly, other studies reported that ED time-based targets resulted in a reduction in LOS in emergency care [19–21] and in-hospital mortality [22, 23]. In Australia, NEAT was found to be associated with an increase in the number of patients treated and discharged from ED within four hours [24, 25] and a decrease in ‘access block’ (the inability of ED patients from accessing inpatient care due to lack of available inpatient beds) [26, 27]. Of these studies, two were conducted in the state of Victoria, with n = 1 and n = 2 hospital sites respectively, and relatively short study periods [25, 26] limiting their generalisability and ability to identify trends over time. To our knowledge, analysis of state-wide EDs in Victoria has not previously been conducted. Therefore, this study aimed to evaluate the impact of government national healthcare reforms on ED time-base process outcomes, in particular those publicly reported on the MyHospitals website—patients waiting time to treatment; treatment within recommended time; and departing ED within four hours of arrival for public hospitals in Victoria, Australia.

## Methods

### Study design

This study is a component of a larger mixed-methods research program aimed at increasing understanding of how public reporting may improve quality of care in public and private hospitals in Australia. Previous components of the research program included examining the perspectives of multiple stakeholders including consumer advocates, providers, purchasers [28], public hospital medical directors[29, 30], general practitioners (GPs) [31] and patients [32]. This component used a quantitative approach to understand the effect of national healthcare reforms on various ED time-based process outcomes.

This study involved an uncontrolled, interrupted time series (ITS) analysis of Victorian ED presentations data. ITS is a powerful quasi-experimental research design for evaluating the effect of an intervention when random allocation is not feasible. ITS contains a series of observations related to the outcome of interest at multiple time points before and after the introduction of an intervention. The trends before and after the intervention are compared to determine the effect of the intervention from its secular (underlying) trend [33–35]. It is particularly useful in the analysis of ‘natural experiments’ in real world settings, for example the introduction of a national policy or incentive.

### Data source

A data request was submitted to the Victorian Department of Health and Human services (DHHS) (data requests activities transferred to The Victorian Agency for Health Information in 2017) for data access to the Victorian Emergency Minimum Dataset (VEMD) [36]. De-identified patient-level data from VEMD spanning years 2006 to 2016 were provided by DHHS. The VEMD records all presentations to EDs in Victorian public hospitals that have a designated 24-hour ED. The VEMD includes de-identified demographic, administrative and clinical data. Data are collected by individual hospitals using standard definitions and protocols, then transferred to the Data Collections Unit (DCU) which manages VEMD operations. Thirty-nine hospitals currently provide data to the VEMD, of those, one hospital provided data only from 2011 onwards (n = 78,139) and therefore was excluded from the analyses.

### Inclusion and exclusion criteria

We selected all ED presentations between 2006 and 2016. Cases were excluded if the: a) type of visit was a planned return visit, pre-arranged admission or patient in transit (n = 314,857); b) patient was dead on arrival (n = 20,274); c) patient did not wait to be attended by a healthcare professional (n = 909,012); c) hospital was others than public acute hospitals (e.g. specialised hospital—women or children hospitals) (n = 1,505,268); or d) waiting time to treatment was greater than eight hours (n = 2,957). With regard to (c), we excluded hospital that had only small numbers of their type. With regard to (d), a wait time more than eight hour was considered a potential data error or may have represented a patient who did not require emergency care.

### Government emergency department targets

Australian national healthcare reforms relevant to this study began in August 2011. The VEMD provided by DHHS did not include the full date of patient presentations, only year of presentations. For these analyses, we defined the pre-reform period as 2006 through to 2010, and the post-reform period as 2011 through to 2016.

### Outcomes measures

Outcomes of interest were waiting time to treatment, treatment within recommended time, and departing ED within four hours of arrival. Waiting time to treatment was defined as the time between a patient’s arrival at the ED and the commencement of their clinical care and measured in minutes. Treatment within recommended time referred to the recommended maximum waiting times for commencement of clinical care based on patient’s urgency need for care. There are five urgency categories defined by the Australasian Triage Scale [16]: 1) resuscitation (immediate treatment–defined as within two minutes in this study [as per the MyHospitals website]); 2) emergency (within 10 minutes); 3) urgent (within 30 minutes); 4) semi-urgent (within 60 minutes); and 5) non-urgent (within 120 minutes). Treatment within recommended time was coded as yes or no and was derived from waiting time to treatment and urgency category variables. Departing ED within four hours of arrival was defined as the time between a patient’s arrival at the ED and their physical departure from the ED. Departing ED within four hours of arrival was coded as yes or no and was derived from length of stay in ED variable.

### Explanatory variables

Gender, age, triage and diagnosis were all based on VEMD variables as defined at the time of the ED presentation. Gender was categorised into three groups: male; female; and intersex. Age was categorised into 11 groups: 0–4; 5–14; 15–24; 25–34; 35–44; 45–54; 55–64; 65–74; 75–84; 85–94; and ≥95. Triage was based on the five Australasian Triage Scale [16] classification categories described above. Diagnosis was based on the best information available after the patient’s ED presentation using the International Classification of Diseases, 10^th^ Revision, Australian Modification (ICD 10-AM) diagnosis codes [37]. Diagnosis was grouped into 23 categories (S1 Text).

### Statistical analyses

Descriptive statistics of key variables were used to characterise the differences before and after the introduction of national healthcare reforms stratified by hospital peer groups. Hospitals were categorised into four groups as defined by the AIHW [38]: 1) major hospitals (principal referral); 2) large metropolitan and regional hospitals (public acute group A); 3) medium metropolitan and regional hospitals (public acute group B); and 4) small hospitals all areas (public acute group C).

Segmented linear regression analyses, adjusted for demographic and clinical factors, were conducted to assess the significance of change in level and slope of the regression lines of waiting time to treatment, before and after the introduction of national healthcare reforms. Interaction effects between triage category and year of presentation were conducted. Similarly, segmented logistic regression analyses, adjusted for demographic and clinical factors, were conducted to estimate treatment within recommended time, and departing ED within four hours of arrival. All models were stratified by hospital peer groups to allow valid comparisons across similar hospitals. Residual analyses were conducted to examine the presence of serial autocorrelation. A p-value of less than 0.05 was considered statistically significant in all analyses. Data analyses were conducted using STATA version 14 (StataCorp, College Station, TX, USA). Ethical approval for this study was obtained from the Melbourne School of Population and Global Health Human Ethics Advisory Group, The University of Melbourne.

## Results

Over the 11-year period 2006–2016, there were 13,241,509 ED presentations in 34 Victorian hospitals. Of these, 3,615,442 (27.30%) presentations were in six major hospitals, 5,786,885 (43.70%) in 14 large hospitals, 3,123,792 (23.59%) in nine medium hospitals, and 715,390 (5.40%) in five small hospitals.

### Patient characteristics

Patient characteristics and ED outcomes for pre and post reform periods, stratified by hospital peer groups are described in Table 1. Across all hospital peer groups and periods, the proportion of presentations by females and males was similar. Presentations were most common among those aged 15–34 years. The majority of presentations were classified as urgent and semi-urgent. There was a decrease in the number of presentations classified as non-urgent in the post reform period.

**Table 1:** Patients characteristics by hospital peer groups (N = 13,241,509).

	Major hospitals (n = 6)		Large hospitals (n = 14)		Medium hospitals (n = 9)		Small hospitals (n = 5)	
	Pre ED targets (n = 1,432,764)	Post ED targets (n = 2,182,678)	Pre ED targets (n = 2,410,379)	Post ED targets (n = 3,376,506)	Pre ED targets (n = 1,346,836)	Post ED targets (n = 1,776,956)	Pre ED targets (n = 315,647)	Post ED targets (n = 399,743)
**Gender**								
*Male*	763,115 (53.26%)	1,137,252 (52.10%)	1,243,068 (51.57%)	1,702,702 (50.43%)	662,934 (49.22%)	856,348 (48.19%)	165,902 (52.56%)	205,857 (51.50%)
*Female*	669,643 (46.74%)	1,045,400 (47.90%)	1,167,310 (48.43%)	1,673,772 (49.57%)	683,900 (50.78%)	920,599 (51.81%)	149,745 (47.44%)	193,881 (48.50%)
*Intersex*	6 (0.00%)	26 (0.00%)	1 (0.00%)	32 (0.00%)	2 (0.00%)	9 (0.00%)	0 (0.00%)	5 (0.00%)
**Age groups**								
*0–4*	93,257 (6.51%)	160,605 (7.36%)	221,202 (9.18%)	289,127 (8.56%)	179,772 (13.35%)	217,976 (12.27%)	28,907 (9.16%)	34,706 (8.68%)
*5–14*	72,076 (5.03%)	126,123 (5.78%)	225,946 (9.37%)	291,386 (8.63%)	169,435 (12.58%)	198,474 (11.17%)	36,691 (11.62%)	42,284 (10.58%)
*15–24*	197,817 (13.81%)	280,195 (15.51%)	362,403 (15.04%)	481,348 (14.26%)	203,781 (15.15%)	254,445 (14.32%)	51,628 (16.36%)	58,920 (14.74%)
*25–34*	217,382 (15.17%)	338,631 (15.51%)	307,695 (12.77%)	433,062 (12.83%)	186,715 (13.86%)	254,956 (14.35%)	36,345 (11.51%)	45,772 (11.45%)
*35–44*	174,804 (12.20%)	263,774 (12.08%)	285,302 (11.84%)	384,380 (11.38%)	166,713 (12.38%)	214,072 (12.05%)	35,082 (11.11%)	40,890 (10.23%)
*45–54*	152,018 (10.61%)	236,105 (10.82%)	252,189 (10.46%)	362,537 (9.72%)	128,480 (9.54%)	177,507 (9.99%)	32,447 (10.28%)	41,053 (10.27%)
*55–64*	142,849 (9.97%)	218,694 (10.02%)	221,310 (9.18%)	328,227 (9.72%)	102,667 (7.62%)	146,859 (7.14%)	30,527 (9.67%)	40,773 (10.20%)
*65–74*	139,835 (9.76%)	209,486 (9.60%)	199,988 (8.30%)	310,045 (9.18%)	79,971 (5.94%)	126,858 (6.36%)	26,064 (8.26%)	40,560 (10.15%)
*75–84*	158,719 (11.08%)	215,308 (9.86%)	217,019 (9.00%)	307,043 (9.09%)	83,191 (6.18%)	112,947 (6.36%)	25,787 (8.17%)	35,256 (8.82%)
*85–94*	76,775 (5.36%)	123,433 (5.66%)	107,941 (4.48%)	175,033 (5.18%)	42,477 (3.15%)	67,391 (3.79%)	11,310 (3.58%)	18,283 (4.57%)
*>95*	7,231 (0.50%)	10,324 (0.47%)	9,384 (0.39%)	14,318 (0.42%)	3,634 (0.27%)	5,471 (0.31%)	859 (0.27%)	1,246 (0.31%)
**Triage**								
*Resuscitation*	22,169 (1.55%)	18,982 (0.87%)	15,443 (0.64%)	18,361 (0.54%)	2,187 (0.16%)	3,950 (0.22%)	708 (0.22%)	978 (0.24%)
*Emergency*	178,028 (12.43%)	275,626 (12.63%)	260,324 (10.80%)	447,624 (13.26%)	82,085 (6.09%)	144,599 (8.14%)	11,873 (3.76%)	26128 (6.54%)
*Urgent*	557,464 (38.91%)	890,902 (40.82%)	809,273 (33.57%)	1,298,446 (38.46%)	368,062 (27.33%)	540,121 (30.40%)	63,401 (20.09%)	111,325 (27.85%)
*Semi-urgent*	578,133 (40.35%)	847,169 (38.81%)	1,054,735 (43.76%)	1,384,240 (41.00%)	746,338 (55.41%)	929,477 (52.31%)	140,062 (44.37%)	180,332 (45.11%)
*Non-urgent*	96,970 (6.77%)	149,999 (6.87%)	270,604 (11.23%)	227,835 (6.75%)	148,164 (11.00%)	158,809 (8.94%)	99,603 (31.56%)	80,980 (20.26%)
**Mean time to treatment in minutes (SD)**	41.82 (58.47)	35.18 (45.79)	40.54 (55.42)	36.64 (49.31)	46.61 (55.89)	41.36 (54.23)	34.89 (44.40)	32.69 (41.51)
**Treatment within recommended time**	1,003,294 (70.03%)	1,598,147 (73.22%)	1,783,998 (74.01%)	2,506,692 (74.24%)	961,104 (71.36%)	1,302,018 (73.27%)	262,753 (83.24%)	335,171 (83.85)
**Departing ED within 4 hours**	747,969 (52.20%)	1,336,706 (61.24%)	1,450,861 (60.19%)	2,112,795 (62.57%)	968,983 (71.95%)	1,255,283 (70.64%)	269,496 (85.38%)	323,262 (80.87%)

ED emergency department; SD standard deviation; Pre ED target period = 2006 to 2010; Post ED target period = 2011 to 2016

Following the introduction of national healthcare reforms, all hospital peer groups showed a decline in the mean waiting time to treatment and an increase in the proportion of patients treated within the recommended time. There was an increase in the proportion of patients who departed ED within four hours of arrival in the post reform period among major and large hospitals, whereas there was a decrease among medium and small hospitals.

### Waiting time to treatment stratified by hospital peer groups

**Fig 1:**
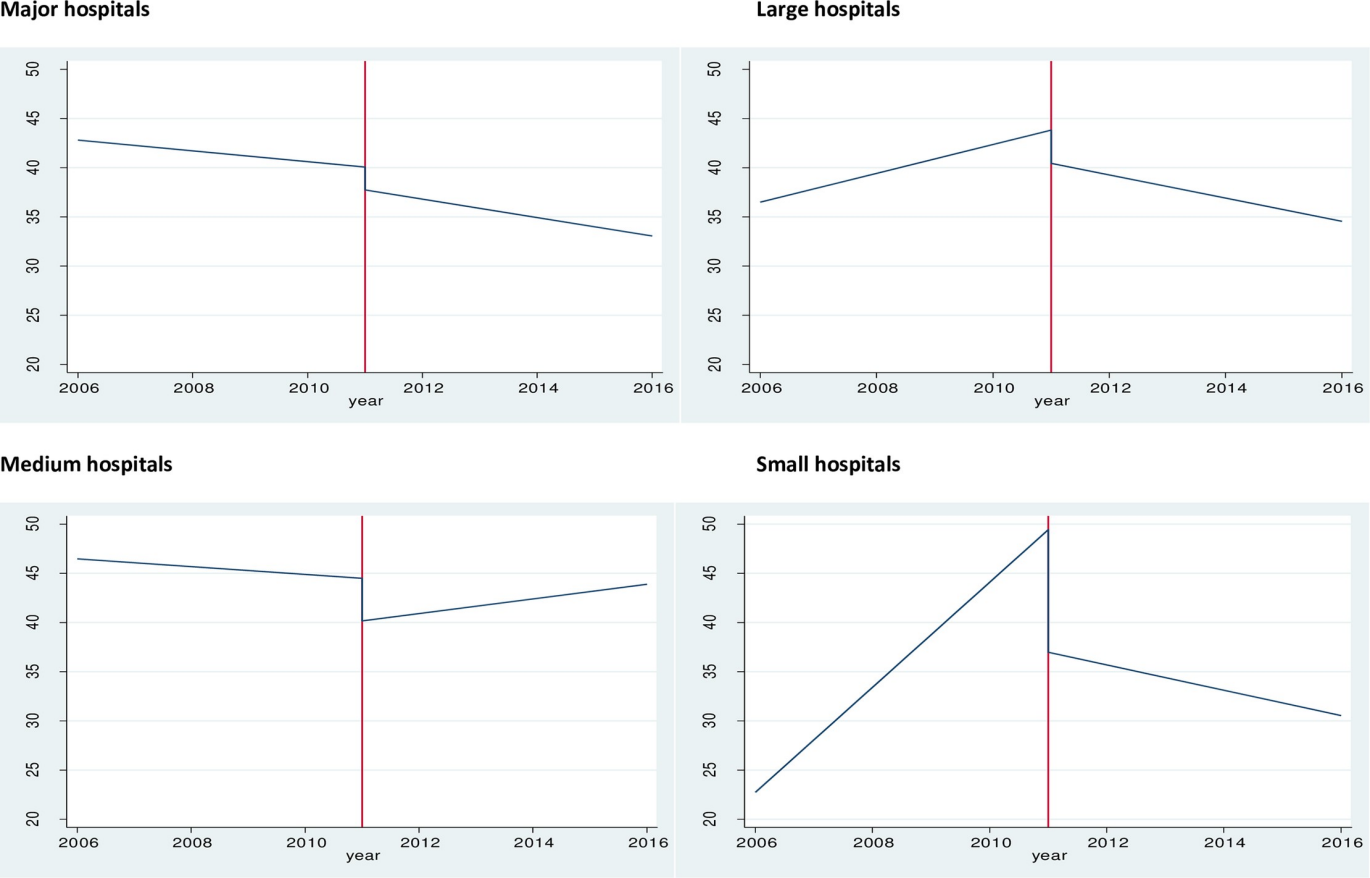
Mean time to treatment (minutes) by year of presentation and hospital peer groups.

Results of the segmented linear regressions for waiting time to treatment, adjusted for gender, age, triage and diagnosis, and stratified by hospital peer groups, are presented in Fig 1 (S1 Table). Immediately after the implementation of national healthcare reforms (as judged by intercepts), waiting times to treatment improved significantly across all hospital peer groups, with the largest improvement observed in small hospitals (-12.44 minutes). In the post reform period, all hospital peer groups continued significant improvements in waiting time to treatment, with the exception of medium hospitals (+1.14 minutes per year compared to the pre-intervention trend).

The largest improvements in waiting time to treatment was observed among small hospitals (-6.62 minutes per year compared to the pre-intervention trend).

### Waiting time to treatment by triage categories and stratified by hospital peer group

**Fig 2:**
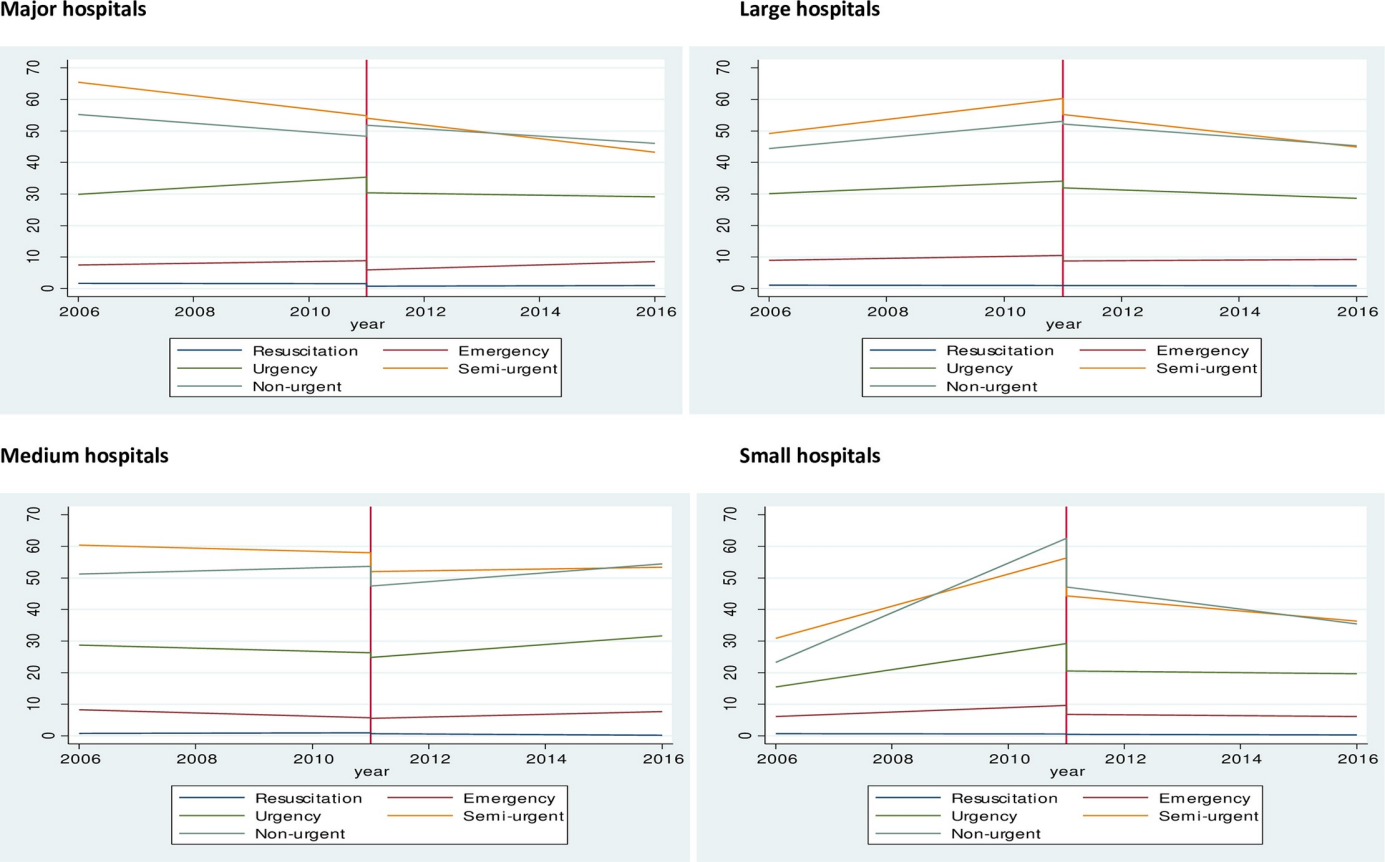
Mean time to treatment (minutes) by triage categories, year of presentation and hospital peer groups.

To assess whether waiting time to treatment differ by triage categories, interaction effects between triage category and year of presentation adjusted for age, gender and diagnosis, and stratified by hospital peer groups were conducted (Fig 2). There were significant triage differences in the trends of waiting time to treatment by hospital peer groups, with the exception of resuscitation. After the implementation of national healthcare reforms, waiting time to treatment was significantly shorter across all triage categories and hospital peer groups (S2 Table), with the exception of the non-urgent category in major hospitals, which showed an immediate increase of 3.53 minutes. In the post reform period, there was a decline in the mean waiting time to treatment for urgent, semi-urgent and non-urgent presentations in major hospitals, large and small hospitals. In contrast, medium hospitals experienced an increase in the mean waiting time to treatment for urgent (+1.38 minutes per year), semi-urgent (+0.26 minutes per year) and non-urgent (+1.42 minutes per year) presentations.

### Treatment within recommended time stratified by hospital peer groups

Results of the segmented logistic regressions for treatment within recommended time adjusted for age, gender and diagnosis, and stratified by hospital peer groups are presented in Table 2. Across all hospital peer groups, the odds of patients being treated within recommended time increased following the implementation of national healthcare reforms. The largest increase was observed among small hospitals (odds ratio (OR) 1.79; 95% confidence intervals (CI) 1.74–1.83). Compared to the pre-intervention trends, the odds of patients being treated within the recommended time in the post-reform period continued to increase in major (OR 1.02; 95% CI 1.01–1.02), large (OR 1.12; 95% CI 1.12–1.12) and small (OR 1.33; 95% CI 1.32–1.35) hospitals. In contrast, patients were less likely to be treated within the recommended time in medium hospitals compared to the pre-intervention trend (OR 0.96; 95% CI 0.96–0.96).

**Table 2:** Segmented logistic regression models of the relations between government targets, treatment within recommended time, and departing emergency department within 4 hours stratified by hospital peer groups.

	Major hospitals (n = 6)			Large hospitals (n = 14)			Medium hospitals (n = 9)			Small hospitals (n = 5)		
	OR	95% CI	p value	OR	95% CI	p value	OR	95% CI	p value	OR	95% CI	p value
**Treatment within recommended time**^**a**^												
*Pre-intervention slope*	0.99	0.99–0.99	0.002	0.93	0.92–0.93	<0.001	1.01	1.01–1.01	<0.001	0.79	0.78–0.79	<0.001
*Change in intercept*	1.13	1.12–1.15	<0.001	1.14	1.13–1.15	<0.001	1.15	1.14–1.17	<0.001	1.79	1.74–1.83	<0.001
*Change in slope*	1.02	1.02–1.02	<0.001	1.12	1.12–1.12	<0.001	0.96	0.96–0.96	<0.001	1.33	1.32–1.35	<0.001
**Departing ED within 4 hours**^**b**^												
*Pre-intervention slope*	0.98	0.98–0.98	<0.001	0.97	0.97–0.97	<0.001	0.93	0.93–0.94	<0.001	0.89	0.88–0.89	<0.001
*Change in intercept*	1.13	1.12–1.15	<0.001	1.03	1.03–1.04	<0.001	0.98	0.97–0.99	0.010	1.08	1.05–1.11	<0.001
*Change in slope*	1.16	1.16–1.17	<0.001	1.16	1.16–1.17	<0.001	1.18	1.17–1.18	<0.001	1.19	1.17–1.20	<0.001

OR odds ratio; CI confidence intervals

^a^models adjusted for gender, age and diagnosis

^b^models adjusted for gender, age, triage category and diagnosis

### Departing emergency department within four hours of arrival stratified by hospital peer groups

Results of the final segmented logistic regressions for patients departing ED within four hours of arrival, adjusted for age, gender, triage and diagnosis, and stratified by hospital peer groups, are presented in Table 2. Following the implementation of national healthcare reforms, the odds of patients departing ED within four hours of arrival increased across all hospital peer groups, except for medium hospitals (OR 0.98; 95% CI 0.97–0.99). In the post reform period, across all hospital peer groups, patients were likely to depart ED within four hours of arrival.

## Discussion

### Main findings

This natural experiment study investigated the impact of national healthcare reforms on ED time-based process outcomes in Victoria, Australia. Our findings suggest that reforms influenced ED time-based process outcomes, but the recommended ED time-based targets were generally not achieved. Following implementation of reforms, waiting time to treatment, treatment within recommended time, and departure within four hours of arrival improved immediately across all hospital peer groups. Long-term improvements for waiting time to treatment, and treatment within the recommended time, were observed across all hospital peer groups with the exception of medium hospitals. Departure within four hours of arrival also exhibited long-term improvements across all hospital peer groups. ED waiting times to treatment varied across triage categories.

Improvement in the timeliness of accessing emergency care and the LOS following the introduction of national healthcare reforms varied across hospital peer groups, with the greatest improvement in small hospitals. This is consistent with previous Australian research which showed that small hospitals have the shortest waiting time to treatment [39] and patients were more likely to depart the ED within four hours of arrival than in major, large and medium hospitals [40]. Such improvements are likely to be dependent on individual hospitals’ behavioural responses to government targets and initiatives undertaken to address them. It is unclear how small hospitals improved their ED time-based process outcomes; particularly as NEAT does not provide specific guidance on change processes. Instead, individual hospitals are responsible for the development and implementation of change processes based on their own perceived issues. A review by Crawford et al. [41] described a number of initiatives implemented in Australia to address ED time-based process targets, including waiting room nurses, streaming (directing patient flow based on illness or injury severity), rapid assessment teams, short stay units, and care coordination programs, but the authors did not differentiate between hospital types. Future research investigating ED initiatives to reduce patient waiting times and LOS in small hospitals should seek to provide insights into successful practices which could be applied in other hospitals.

The study was not designed to determine why and how hospitals improved or worsened their performance in the post reform period. Therefore, it is not known why the results of medium hospitals worsened compared to other hospital peer groups. Following the introduction of national healthcare reforms, patients presenting to medium hospitals waited an additional 1.14 minutes per year and 4% were less likely to be treated within recommended time compared to the pre-intervention trends. It is unclear if these results are clinically important as we did not have patient’s outcomes measures. A case study evaluation of an Australian regional hospital clinical redesign activities following the reforms suggested that targets were important drivers for improvement in ED access, however not at the expense of patient safety [42]. The authors identified strong clinical leadership, support from management, and investment into infrastructure and workforce as essential for improvement in ED access [42]. Similarly, our previous qualitative study with medical directors of public hospitals in Victoria highlighted lack of leadership from government and within hospitals (i.e. boards and executives) and lack of resources (i.e. staff expertise, sufficient staff numbers, existing data) as barriers to quality improvement within hospitals [30]. Further research at individual hospital level is required to identify change processes that lead to or impede improvements in ED performance.

The timeliness of accessing emergency care improved significantly for all triage categories, with the exception of resuscitation. As expected, there was minimal to no effects of national healthcare reforms on waiting time to treatment among resuscitation across all hospital peer groups, which suggests that patients were treated within an appropriate time frame before and after the introduction of reforms. Semi-urgent and non-urgent presentations (also referred to as GP-type presentations) exhibited the greatest reduction in waiting time to treatment, in particular among small hospitals. Of note, the proportion of semi-urgent and non-urgent presentations were greatest among small hospitals, whereas, as expected, major and large hospitals had a greater proportion of emergency and urgent presentations. Green et al. [43] have similarly found that smaller Australian hospitals, which have greater volume of non-urgent patients, have better wait time performance than larger Australian hospitals which proportionally have far lower numbers of non-urgent patients.

Despite significant improvements in ED time-based process outcomes following the introduction of national healthcare reforms, ED time-based targets of 90% of patients being treated and departing the ED within 4 hours, were generally not met across any of the hospital peer groups. Less than 80% of patients classified as urgent and semi-urgent were seen within the recommended time. This is consistent across Australia [40, 44] and internationally [45, 46]. The 4-hour rule was implemented with a limited evidence base [47] and debate surrounding its suitability and sustainability continues [48].

Past research suggests that publicly reporting ED time-based targets may results in perverse incentives, such as gaming of the system by increasing hospital in-patient admission rates and therefore reducing ED LOS [49]. We did not evaluate these unintended consequences. Further research is warranted to assess whether the reductions apparent in ED LOS are associated with increased admissions rates. Perhaps as a response to the 4-hour rule and unattainable 90% target, NEAT has recently been abolished by the Australian government [50], but it has remained a key ED performance indicator on the MyHospitals [15] and some states performance websites [51, 52]. Research also recommends the inclusion of patients’ ED clinical outcomes alongside ED time-based targets to minimise perverse incentives and unintended consequences [10, 53]. Clinical outcomes of patients in emergency care are currently not publicly reported in Australia.

### Strengths and limitations

By employing ITS, a powerful quasi-experimental research design, evaluation of the effect of national healthcare reforms on ED time-based process outcomes was possible. The analyses included state-wide population coverage of ED presentations over a period of 11 years, allowing identification of secular trends. Multilevel analysis, adjusting for clustering at the hospital level, were initially conducted, however, the model showed low intra class correlation of 2% (i.e. variation attributed to the hospital). Analyses were instead conducted separately for each hospital peer group to ensure equitable comparisons across hospitals types.

Limitations of the study included confounding influences making it difficult to attribute the observed changes to a specific intervention [33]. For example, financial incentives for achieving ED targets were introduced concurrently with public reporting. However, most states and territories were unable to reach the ED targets over the study period [40, 44], therefore it is unlikely that financial incentives had a substantial impact on ED time-based process outcomes. Nevertheless, given that financial incentives were removed in 2014, there is an opportunity to evaluate and isolate the effects of public reporting from financial incentives on ED time-based process outcomes when sufficient data points are available. Introducing a suitable comparator group may alleviate misattribution of cause. However, we were unable to select a comparator group given that the healthcare reforms were implemented nationally. Finally, improvement in ED time-based process outcomes does not necessarily represent an improvement in patient care. We did not objectively measure clinical outcomes of patients; therefore, we are unable to comment on whether process improvements led to better quality of care and patient outcomes. Future research is warranted to better understand the relationships between ED targets, ED time-based process measures and patient clinical outcomes.

### Conclusions

Australian national healthcare reforms appear, in the state of Victoria, to have encouraged improved ED patient waiting times, treatment within recommended time, and departure within four hours of arrival. Despite these improvements, the reforms were not able to stimulate attainment of the recommended Australian national targets. ED performance varied widely depending on hospital size, with small hospitals significantly improving their performance after the introduction of the reforms. The study highlights the potential for nationally implemented healthcare system reforms to prompt fundamental changes in ED processes, despite being linked to targets that proved to be generally unattainable. The variation across triage categories and hospital peer groups provides unique future opportunities to understand behavioural change at the hospital level toward quality and safety improvement.

## Supporting information

S1 Table Segmented linear regression models of the relations between government targets and waiting time to treatment stratified by hospital peer groups. (DOCX)

S2 Table Segmented linear regression models of the relations between government targets, triage categories and waiting time to treatment stratified by hospital peer groups. (DOCX)

S1 Text Diagnosis group. (DOCX)
