# Supplementary material for: The impact of Australian healthcare reforms on emergency department time-based process outcomes: An interrupted time series study
Source: PLoS One. 2018 Dec 12;13(12):e0209043. doi: 10.1371/journal.pone.0209043 (PMC6291126; doi:10.1371/journal.pone.0209043)
Supplement: S1 Table — (DOCX) [file pone.0209043.s001.docx]

**Segmented linear regression models of the relations between government targets and waiting time to treatment stratified by hospital peer groups**

|  | **Major hospitals (n=6)** | | | **Large hospitals (n=14)** | | | **Medium hospitals (n=9)** | | | **Small hospitals (n=5)** | | |
| --- | --- | --- | --- | --- | --- | --- | --- | --- | --- | --- | --- | --- |
|  | **β** | **95% CI** | **p value** | **β** | **95% CI** | **p value** | **β** | **95% CI** | **p value** | **β** | **95% CI** | **p value** |
| **Pre-intervention slope** | -0.54 | -0.61 - -0.49 | <0.001 | 1.46 | 1.42-1.51 | <0.001 | -0.39 | -0.46 - -0.33 | <0.001 | 5.34 | 5.23-5.44 | <0.001 |
| **Change in intercept** | -2.33 | -5.54 - -2.11 | <0.001 | -3.36 | -3.54 – -3.19 | <0.001 | -4.35 | -4.60 - -4.10 | <0.001 | -12.44 | -12.84 - -12.03 | <0.001 |
| **Change in slope** | -0.39 | -0.45 - -0.32 | <0.001 | -2.65 | -2.70 - -2.60 | <0.001 | 1.14 | 1.07-1.22 | <0.001 | -6.62 | -6.75 - -6.50 | <0.001 |

All models adjusted for gender, age, triage category and diagnosis
