# Supplementary material for: The impact of Australian healthcare reforms on emergency department time-based process outcomes: An interrupted time series study
Source: PLoS One. 2018 Dec 12;13(12):e0209043. doi: 10.1371/journal.pone.0209043 (PMC6291126; doi:10.1371/journal.pone.0209043)
Supplement: S2 Table — (DOCX) [file pone.0209043.s002.docx]

**Segmented linear regression models of the relations between government targets, triage categories and waiting time to treatment stratified by hospital peer groups**

|  | **Major hospitals (n=6)** | | | **Large hospitals (n=14)** | | | **Medium hospitals (n=9)** | | | **Small hospitals (n=5)** | | |
| --- | --- | --- | --- | --- | --- | --- | --- | --- | --- | --- | --- | --- |
|  | **β** | **95% CI** | **p value** | **β** | **95% CI** | **p value** | **β** | **95% CI** | **p value** | **β** | **95% CI** | **p value** |
| **Pre-intervention slope** |  |  |  |  |  |  |  |  |  |  |  |  |
| *Resuscitation* | -0.01 | -0.05 – 0.03 | 0.532 | -0.01 | -0.04 – 0.02 | 0.493 | 0.04 | -0.07 – 0.16 | 0.454 | -0.04 | -0.12 - -0.05 | 0.416 |
| *Emergency* | 0.27 | 0.23 – 0.32 | <0.001 | 0.31 | 0.27 – 0.35 | <0.001 | -0.51 | -0.56 - -0.45 | <0.001 | 0.70 | 0.56 – 0.84 | <0.001 |
| *Urgency* | 1.10 | 1.03 – 1.18 | <0.001 | 0.78 | 0.71 – 0.85 | <0.001 | -0.49 | -0.57 – -0.41 | <0.001 | 2.74 | 2.60 – 2.89 | <0.001 |
| *Semi-urgent* | -2.14 | -2.26 - -2.02 | <0.001 | 2.21 | 2.13 – 2.30 | <0.001 | -0.49 | -0.59 - -0.39 | <0.001 | 5.09 | 4.91 – 5.26 | <0.001 |
| *Non-urgent* | -1.39 | -1.67 - -1.11 | <0.001 | 1.74 | 1.59 – 1.88 | <0.001 | 0.47 | 0.27 – 0.68 | <0.001 | 7.86 | 7.65 – 8.06 | <0.001 |
| **Change in intercept** |  |  |  |  |  |  |  |  |  |  |  |  |
| *Resuscitation* | -0.79 | -0.95 - -0.63 | <0.001 | -0.02 | -0.13 – 0.10 | 0.774 | -0.27 | -0.75 – 0.20 | 0.264 | -0.06 | -0.41 – 0.29 | 0.732 |
| *Emergency* | -2.93 | -3.11 - -2.74 | <0.001 | -1.71 | -1.86 - -1.56 | <0.001 | -0.18 | -0.36 – 0.01 | 0.064 | -2.75 | -3.27 – 2.24 | <0.001 |
| *Urgency* | -5.03 | -5.33 - -4.73 | <0.001 | -2.10 | -2.37 - -1.84 | <0.001 | -1.52 | -1.83 - -1.20 | <0.001 | -8.67 | -9.23 - -8.10 | <0.001 |
| *Semi-urgent* | -0.76 | -1.21 - -0.31 | 0.001 | -5.02 | -5.35 - -4.69 | <0.001 | -5.90 | -6.30 - -5.51 | <0.001 | -12.06 | -12.79 - -11.32 | <0.001 |
| *Non-urgent* | 3.53 | 2.50 - 4.56 | <0.001 | -0.91 | -1.55 – -0.27 | 0.006 | -6.22 | -7.08 - -5.36 | <0.001 | -15.47 | -16.45 - -14.49 | <0.001 |
| **Post-intervention slope** |  |  |  |  |  |  |  |  |  |  |  |  |
| *Resuscitation* | 0.05 | 0.02 – 0.08 | 0.004 | -0.02 | -0.04 – 0.01 | 0.177 | -0.12 | -0.18 - -0.06 | 0.001 | -0.05 | -0.11 – 0.02 | 0.149 |
| *Emergency* | 0.53 | 0.50 – 0.55 | <0.001 | 0.08 | 0.06 – 0.11 | <0.001 | 0.44 | 0.40-0.47 | <0.001 | -0.15 | -0.21 - -0.09 | <0.001 |
| *Urgency* | -0.25 | -0.30 - -0.21 | <0.001 | -0.66 | -0.70 - -0.61 | <0.001 | 1.38 | 1.32-1.44 | <0.001 | -0.16 | -0.25 - -0.08 | <0.001 |
| *Semi-urgent* | -2.16 | -2.23 - -2.09 | <0.001 | -2.07 | -2.13 - -2.02 | <0.001 | 0.26 | 0.19-0.33 | <0.001 | -1.59 | -1.72 - -1.46 | <0.001 |
| *Non-urgent* | -1.15 | -1.32 – -0.99 | <0.001 | -1.38 | -1.51 - -1.25 | <0.001 | 1.42 | 1.24-1.59 | <0.001 | -2.33 | -2.53 - -2.14 | <0.001 |

All models adjusted for gender, age and diagnosis
