# Supplementary material for: The impact of Australian healthcare reforms on emergency department time-based process outcomes: An interrupted time series study
Source: PLoS One. 2018 Dec 12;13(12):e0209043. doi: 10.1371/journal.pone.0209043 (PMC6291126; doi:10.1371/journal.pone.0209043)
Supplement: S1 Text — (DOCX) [file pone.0209043.s003.docx]

**Diagnosis group**

1. alcohol/drug abuse and alcohol/drug induced mental disorders;
2. blood/immune system illness;
3. circulatory system illness;
4. digestive system illness;
5. gynaecological illness/male reproductive system illness;
6. hepatobiliary system illness;
7. illness of other or unknown systems;
8. illness of the ENT;
9. illness of the eyes;
10. illness of the skin, subcutaneous tissue, breast;
11. injury single/multiple sites, minor and poisoning comatose/conscious;
12. musculoskeletal/connective tissue illness;
13. neurological illness;
14. newborn/neonate;
15. obstetric illness;
16. other presentation;
17. psychiatric illness;
18. respiratory system illness;
19. social problem;
20. system infection/parasites;
21. urological illness;
22. allergy;
23. endocrine, nutritional and metabolic system.
